# Induction and differential expression of certain novel proteins in *Anabaena* L31 under UV-B radiation stress

**DOI:** 10.3389/fmicb.2015.00133

**Published:** 2015-02-24

**Authors:** Piyoosh K. Babele, Garvita Singh, Ashok Kumar, Madhu B. Tyagi

**Affiliations:** ^1^School of Biotechnology, Banaras Hindu UniversityVaranasi, India; ^2^Botany Section, Mahila Maha Vidyalaya, Banaras Hindu UniversityVaranasi, India

**Keywords:** UV-B radiation, cyanobacteria, *Anabaena* L31, 2-D gel electrophoresis, hypothetical proteins

## Abstract

For examining how UV-B radiation alters the proteome of the N_2_-fixing cyanobacterium, *Anabaena* L31, we extracted proteins from cultures irradiated with UV-B + white light and controls (white light irradiated) and analyzed the proteins using two-dimensional gel electrophoresis and matrix-assisted laser desorption/ionization time of flight mass spectrometry (MALDI-TOF MS). Twenty one proteins, including two hypothetical proteins (HPs) were identified and placed in eight functional categories. However several of the proteins were housekeeping proteins involved in key metabolic processes such as carbon, amino acid biosynthesis and energy metabolism, certain proteins seem to have a role in stress (antioxidative enzymes), translation, cellular processes and reductases. Two novel HPs (all3797 and all4050) were characterized in detail. These two were over-expressed after UV-B irradiation and characterized as FAS 1 (all3797) and PRC barrel-like (all4050) proteins. Bioinformatics analysis revealed that the genes of both the HPs have promoter regions as well as transcription binding sites in their upstream region (UTR). Promoters present in all3797 genes suggest their crucial role against UV-B and certain other abiotic stresses. To our knowledge these novel proteins have not been previously reported in any *Anabaena* strains subjected to UV-B stress. Although we have focused our study on a limited number of proteins, results obtained shed light on the highly complicated but poorly studied aspect of UV-B radiation-mediated changes in the proteome and expression of proteins in cyanobacteria.

## INTRODUCTION

While sunlight provides the energy for life, the ultraviolet-B (UV-B; 280–315 nm) and long-wavelength UV light (UV-A; 315–400 nm) components of the solar spectrum are harmful to many organisms. The rise of solar UV-B radiation reaching the earth’s surface due to the depletion of the stratospheric ozone layer caused by the anthropogenic inputs of chlorinated fluorocarbons has become an important issue over the past two decades (Crutzen, 1992; Kerr and McElroy, 1993). UV-B radiation can damage key biological molecules such as proteins, DNA and lipids (Häder et al., 2007; Castenholz and Garcia-Pichel, 2012). Cyanobacteria are photosynthetic prokaryotes with an evolutionary history that precedes the development of atmospheric ozone protection. They grow in a wide range of diverse and extreme habitats such as hot springs, arctic regions, desert soils, and rocky surfaces (Whitton and Potts, 2000). They show considerable metabolic plasticity and, besides assimilating CO_2_ and O_2_, certain species also assimilate N_2_ and H_2_ (Stewart, 1980). Studies conducted so far suggest that the deleterious effects of UV-B radiation in cyanobacteria are in part due to the direct effects on membrane components, growth and survival, motility, pigmentation, uptake of CO_2_, photosynthetic apparatus particularly the reaction center of photosystem II, RuBisCO, N_2_ fixation, DNA, proteins, and enzymes (Wilson et al., 1995; Kumar et al., 2003, 2004; Singh et al., 2010, 2013).

The molecular mechanisms of various physiological responses in cyanobacteria under UV-B radiation stress are poorly explored (Häder et al., 2007; Singh et al., 2010). In general, studies mainly focused on DNA repair and D1 reaction center protein of photosystem II (Vass et al., 2005; Castenholz and Garcia-Pichel, 2012). SDS-PAGE analysis of whole cell protein of certain cyanobacteria under the stress of UV-B radiation showed negligible change in the protein pattern (Chauhan et al., 1998). However, studies on the global gene expression profile under UV-B stress revealed significant alteration within 2 h of exposure (Huang et al., 2002). Transcriptome analysis pointed to changes of several antioxidative genes but the mRNA abundance did not match with the protein level (Gygi et al., 1999). While these reports do shed light on molecular response, there is a general consensus that the study of differential gene expression is only an indirect approach to understanding the molecular mechanisms of stress response including UV-B radiation (Vass et al., 2005; Fulda et al., 2006; Kurian et al., 2006). During the last two decades, proteome analysis employing 2-dimensional gel electrophoresis (2-DE) has become a powerful tool for visualizing many proteins synthesized in the cell and paved the way for understanding global changes in the gene expression (Godon et al., 1998; Hecker and Volker, 2004; Lei et al., 2005; Kumar et al., 2014). A few workers have studied proteomic changes following UV-B irradiation to cyanobacteria and reported significant alterations in the number and expression of proteins (Ehling-Schulz et al., 2002; Gao et al., 2009; Rai et al., 2013). Induction of early shock proteins and late acclimation proteins following UV-B exposure to *Nostoc commune* has been reported (Ehling-Schulz et al., 2002). Similarly, short-term and long-term exposure of UV-B to *Synechocystis* sp. PCC 6803 showed two groups of proteins, namely short-term and long-term responsive proteins on the basis of their expression (Gao et al., 2009). Till date the best characterized cyanobacterial response to UV-B stress is the cyanobacterium *Synechocystis* sp. PCC 6803, and only sporadic reports are available for the N_2_-fixing species from tropical rice paddy fields. It is also pertinent to mention that in most previous studies, the response of cyanobacteria was tested by exposing cultures to UV-B radiation alone, little, if any, attempt has been made to examine such effects in combination with white light.

Prompted by the above lacuna, we examined the effects of UV-B radiation in combination with white (visible) light in the N_2_-fixing cyanobacterium *Anabaena* strain L31. We aimed to (a) examine the changes in proteome under UV-B + white light stress, (b) identify and characterize the novel proteins, and (c) assess the possible role of identified proteins. This study may provide new insights for understanding how *Anabaena* L31 cells can induce certain novel proteins and adapt to UV-B stress via expression of UV-B responsive genes.

## MATERIALS AND METHODS

### TEST ORGANISM AND GROWTH CONDITIONS

The test organism, a filamentous heterocystous cyanobacterium, *Anabaena* strain L31 was obtained from Dr. S. K. Apte, Bhabha Atomic Research Centre, Trombay, Mumbai, India. Axenic cultures were routinely grown diazotrophically in BG 11 medium (Rippka et al., 1979) in a culture room at 27 ± 2°C and illuminated with white light from Sylvania 40 W T12 cool fluorescent tubes at an intensity of 14.4 W m^-2^ for a 14/10 h light/dark cycle. Unless otherwise stated, all the experiments were performed with exponentially grown cultures having an initial dry weight of 0.1 mg mL^-1^.

### SOURCE AND MODE OF UV RADIATION

The source of artificial UV-B irradiation was a UV-B lamp (Cat No. 3-4408, Fotodyne Inc., USA) giving its main output at 312.67 nm. The desired intensity of UV-B reaching the test samples was obtained by adjusting the distance between the UV-B lamp and the test samples. UV-B intensity was measured by Black-Ray J-221, Long Wave Ultraviolet Intensity Meter (UVP Inc., San Gabriel, CA, USA). Percent survival following exposure of cultures to UV-B + white light or UV-B alone was determined as described previously (Singh et al., 2013). For proteome analysis, 200 mL of homogeneous culture suspension was equally divided into two sets and transferred to two sterile “Corning” make glass Petri dishes (120 mm). Set one was placed in a specially fabricated UV-chamber and irradiated with UV-B radiation (1 W m^-2^) together with white light (14.4 W m^-2^) for 6, 9, and 12 h. Set two (served as control) was transferred to culture room and exposed to white light (14.4 W m^-2^). The culture suspension was gently stirred magnetically during UV-B irradiation to facilitate uniform exposure. Thirty mL culture suspensions were removed at indicated time intervals from both the sets and immediately processed for protein extraction. Unless otherwise stated, cultures were always exposed to UV-B radiation in combination with white light.

### PROTEIN EXTRACTION

Whole cell protein was extracted as described previously (Kurian et al., 2006). Cells were harvested from UV-B treated and control (untreated) cultures by centrifugation at 10,000 ×*g* for 10 min at 4°C in a Sorvall RC-5B Refrigerated Superspeed Centrifuge (DuPont Instruments, USA). The resulting pellet was suspended in 10 mM HEPES buffer (pH 7.2) supplemented with 1 mM phenylmethylsulfonyl fluoride (PMSF) and cells were broken by vortexing with glass beads (0.17–0.18 mm) at 4°C. Glass beads and unbroken cells were removed by centrifugation at 10,000 ×*g* for 20 min. The supernatant obtained mainly contained cytoplasmic proteins. Salts and insoluble impurities present, if any, were removed by a 2-D Clean-Up kit (GE Healthcare, UK). The protein content in each sample was measured by Bradford (1976) method.

### PROTEIN SEPARATION BY 2-DIMENSIONAL GEL ELECTROPHORESIS

The first dimension isoelectric focusing (IEF) was performed using Immobiline DryStrip (pH 4–7, 13 cm; GE Healthcare Bio-Sciences AB, Sweden). Protein sample (500 μg) dissolved in rehydration buffer (7 M urea, 2 M thiourea, 2% (w/v) CHAPS, 0.3% (w/v) DTT, and 0.5% (v/v) IPG buffer) was loaded onto each IPG strip and rehydrated overnight at 4°C in the dark. After rehydration, IEF was performed using the Ettan IPGphor 3 IEF system (GE Healthcare, UK) using the following steps; 2 h at 100 V, 2 h at 200 V, 2 h at 500 V, 2 h at 1500 V, 2 h at 3000 V, 2 h at 6000 V, and 8000 V until a total of 60000 Vh was reached. Following the two steps of equilibrations (Nouwens et al., 2002), the second dimension separation was carried out on 12% SDS-PAGE gel at 20 mA/gel using SE 600 Ruby multiple gel-electrophoresis system (GE Healthcare, UK). Three gels were run for each protein sample of UV-B treated and untreated control cultures. Gels were stained with Colloidal Coomassie Blue G-250 (Candiano et al., 2004) and images were captured using the AlphaImager Gel Documentation unit (Alpha Innotech, USA).

### 2-DE GEL ANALYSIS

The gels were scanned and protein spots analyzed by PDQuest software version 8.0.1 (BioRad Laboratories, USA) for spot detection, quantification, background substraction, and spot matching between various gels. Spot quantification in the control and treated gels (UV-B exposed cultures) was performed by spot volumes (intensity × mm^2^) as described by Agrawal et al. (2014). The relative spot volume with respect to the UV-B treated and untreated control samples at desired time were compared using Student’s *t*-test. *P* values less than 0.05 were considered statistically significant.

### PROTEIN SPOT DIGESTION AND MASS SPECTROMETRY

Desired spots were excised from the gel by manual picking using sterile OneTouch spot picker and placed into separate microcentrifuge tubes. MALDI-TOF MS analysis of protein spots was got done commercially from the Centre for Genomic Application (TCGA), New Delhi.

### DATABASE SEARCH AND BIOINFORMATICS ANALYSIS

The proteins were identified by comparing peptide mass fingerprints at the NCBInr database using the Mascot search engine^1^. Search parameters allowed for oxidized methionines, carbamidomethylation of cysteines, and one missed cleavage site of trypsin with mass accuracy of ±30 ppm. Identification was based on the first ranking result and Mascot scores of >74 which indicated that the hits were significant. Physico-chemical properties of proteins were determined using the ProtParam tool^2^ and database of *Nostoc* sp. PCC 7120 as reference (Kaneko et al., 2001). KEGG BLAST search^3^,^4^,^5^ was used to identify the signal transduction pathway for hypothetical proteins (HPs). SignalP 4.0 server^6^ was used for the prediction of putative signal peptides and their cleavage sites. The transmembrane helices and topology of proteins were predicted using the HMMTop^7^ server. InterProScan (version 4.8) server^8^ was used for the elucidation of protein family. Similarity search of HPs against different species from NCBI (National Center for Biotechnology Information) database was done using BLAST (Basic Local Alignment Search Tool) server^9^. Tree was constructed by UPGMA (Unweighted Pair Group Method with Arithmetic Mean) method. Proteins similar to the HPs were used as input for motif elucidation (highly conserved regions) by MEME tool^10^. For the prediction of promoter/*cis*-acting elements for HPs (designated as all3797 and all4050), upstream sequences were retrieved using genome blast^11^. Untranslated upstream region was taken as input to detect the promoter/c*is*-acting elements using BPROM tool from Softberry server^12^. Post-translational modification sites were detected by CBS prediction server^13^. Protein structure modeling and stereochemical properties of HP were done by CPHModels^14^ and PDBSum ^15^.

## RESULTS

### EFFECTS OF UV-B RADIATION ON GROWTH AND SURVIVAL

**FIGURE 1:**
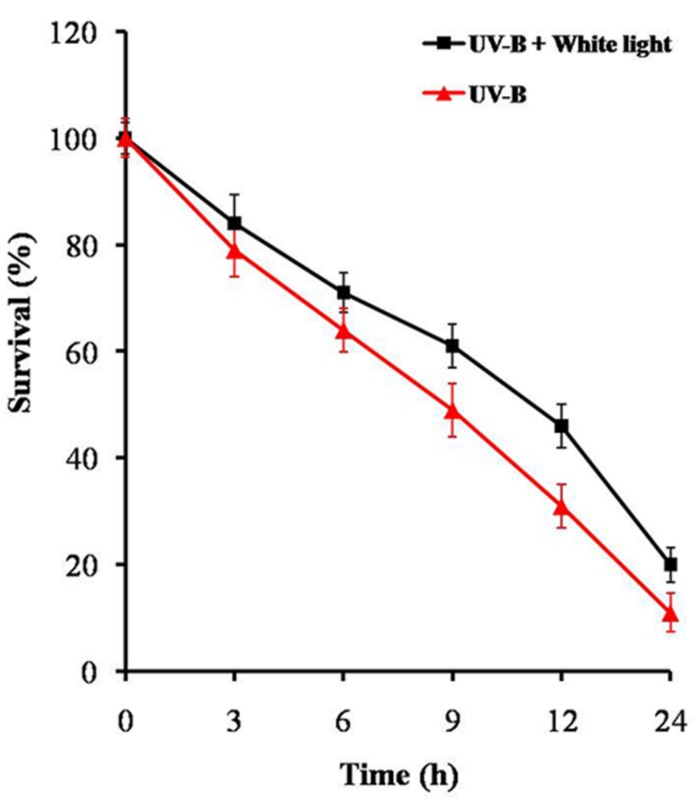
**Percent survival of *Anabaena* L31.** An exponentially grown culture was exposed to UV-B (1 W m^-2^) + white light or UV-B (1 W m^-2^) alone for desired time period and thereafter survival was determined. Results are based on the average of three experiments performed independently under identical conditions.

Before assessing the effects of UV-B radiation + white light on proteome changes, we examined its effect on percentage survival and chlorophyll-a content in *Anabaena* L31. Cells exposed to UV-B (1 W m^-2^) + white light for 9 h showed 61% survival, and decreased to 4% after continuous exposure for 24 h (**Figure 1**). Chlorophyll-a content also decreased by 35% after 9 h of treatment but there was a steep decline after 12 h. This suggests that the cells of *Anabaena* L31 exposed to UV-B radiation (1 W m^-2^) + white light for 9 h do show significant physiological changes but retain more than 60% survival at this dose. Accordingly, all the following experiments were performed after 9 h employing 1 W m^-2^ of UV-B intensity together with white light.

### EFFECTS OF UV-B RADIATION ON PROTEOME

**FIGURE 2:**
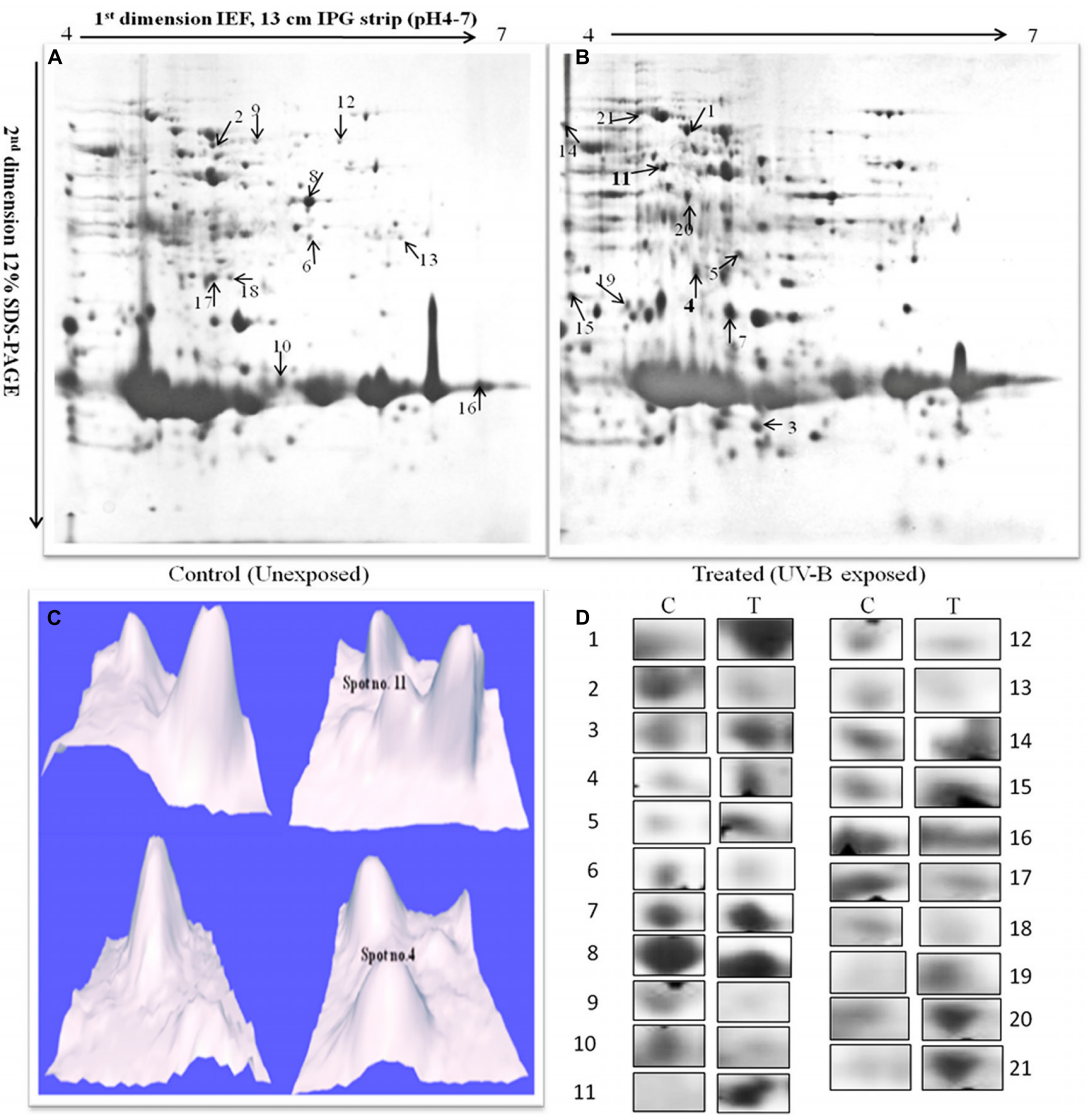
**2-DE images of cytosolic protein from *Anabaena* L31. (A)**-control, and **(B)**-irradiated with UV-B (1 W m^-2^) + white light for 9 h. Proteins were extracted and separated by 2-DE and visualized by CBB staining. 500 μg proteins were loaded on to pH 4–7 IPG dry strips for first dimension IEF followed by 12% linear vertical SDS-PAGE as the second dimension. **(C)**-3-D images of the hypothetical protein spots shown in bold numbers on 2-DE gels, and **(D)**-expression pattern of selected proteins. Lane: C-control, and T- 9 h of UV-B treatment. Numbers represent the spots as indicated on 2-DE gels. Proteins showing significant and reproducible changes were subjected to MALDI-TOF-MS.

Once it became evident that UV-B (1 W m^-2^) + white light treatment do affect survival of *Anabaena* L31, we determined its possible effects on total proteome by employing 2-D gel electrophoresis. Analysis of whole cell protein in gels revealed 210 spots in untreated control and 223 in UV-B-treated *Anabaena* L31 (**Figures 2A,B**). Besides some changes in total number of spots, analysis of gels showed differential expression of several spots in UV-B treated cultures. Henceforth, attention was focused on the protein spots which were showing up-or down regulation on the basis of spot-to-spot comparison. Twenty one protein spots showing more than 4-fold higher and/or lower level of expression in UV-B-treated cultures as compared to untreated control (*p* < 0.05) were selected for MALDI-TOF MS analysis. Of the 21 identified proteins, 11 belonged to up-regulated group including two HPs (**Figure 2C**) and 10 to down-regulated group. **Figure 2D** represents expression level of all the above proteins.

### IDENTIFICATION AND FUNCTIONAL CLASSIFICATION OF DIFFERENTIALLY EXPRESSED PROTEINS

In functional studies we compared the protein sequences with the secondary (or derived) protein databases that contain information on motifs, signature, and protein domains. Highly significant hits against different protein databases allowed us to derive the biochemical functions of query protein. **Table 1** summarizes the key findings related to cyanobacterial processes responding to UV-B treatment based on the identification of representative proteins. All the 21 proteins identified in this study were sorted according to the functional categories defined by cyanobase. On the basis of physiological functions, these proteins were placed in eight functional groups which included carbohydrate metabolism, photosynthesis and respiration, energy metabolism, translation and cellular processes, amino acid biosynthesis, hypothetical, reductases, chaperone, and antioxidative enzymes (**Table 1**). Notably, two HPs namely, all3797 and all4050 were found among the 21 identified proteins.

**Table 1:** Differentially expressed proteins identified in the cellular soluble fraction of *Anabaena* L31 by MALDI-TOF MS.

Spot no.	Proteins	ORF	Gene ID	Length (amino acid)	Mass	PI	Mascot score	Functional domain (InterPro Scan)	E.C. no./pathway	Expression level
**Carbohydrate metabolism**
2	Phosphoglucomutase/phosphomannomutase	all3964	17231542	475	51819.9	5.1	160	IPR005841 Alpha-D-phosphohexomutase_SF	5.4.2.8	Down
8	Fructose-1,6-bisphosphate aldolase	all4563	17232055	359	38616.7	5.4	82.4	IPR000771 Ketose_bisP_aldolase_II	4.1.2.13	Down
9	Glucose-6-phosphate isomerase	alr1050	17228545	528	57818.9	5.3	125	IPR001672 G6P_isomerase	5.3.1.9	Down
**Energy metabolism (photosynthesis and respiration)**
17	F0F1 ATP synthase subunit beta	all5039	17232531	482	52016.3	4.9	143	IPR000194 ATPase_F1/V1/A1_a/bsu_nucl-bd	3.6.3.14	Down
18	Phosphoribulokinase	alr4123	17231615	334	38324	5.5	150	IPR006082 Phosphoribulokinase	2.7.1.19	Down
19	Inorganic pyrophosphatase	all3570	17231062	169	18960.6	4.5	99.8	IPR008162 Pyrophosphatase	3.6.1.1	Up
10	apcF gene product	all2327	17229819	169	18505	5.5	478	IPR006245 Allophycocyanin beta subunit	Photosynthesis antenna protein	Down
16	Phycocyanin A subunit	alr0529	61658473	91	9756.9	10	101	IPR009050 Globin-like	Photosynthesis antenna protein	Down
13	Phycoerythrocyanin alpha chain	alr0524	17228020	162	17454.5	6.4	83.9	IPR012128 Phycobilisome_asu/bsu	Photosynthesis antenna protein	Down
**Chaperones**
1	Chaperonin GroEL	all3662	17231289	544	57951.2	4.9	195	IPR001844 Chaperonin_Cpn60	RNA degradation	Up
3	groES gene product	alr3661	17231153	103	10739	5.2	317	IPR011032 GroES-like	RNA degradation	Up
**Reductases**
15	Heterocyst ferredoxin	all1430	17228925	98	10818	4.3	177	IPR001041 2Fe–2S ferredoxin type domain	Photosynthesis electron carrier	Up
20	1-Cys peroxiredoxin	alr4404	75907581	212	23755.8	5	99.2	IPR000866 AhpC/TSA	1.11.1.15	Up
7	Iron superoxide dismutase	alr2938	17230430	200	22383.9	5.2	98.7	IPR001189 Mn/Fe_SOD	1.15.1.1	Up
**Translation and cellular processes**
14	30S ribosomal protein S1	all0136	17227632	343	38232	4.6	477	IPR000110 Ribosomal protein S1	Translation initiation	Up
21	DNA-directed RNA polymerase subunit alpha	all4191	17231683	315	35072.6	4.7	132	IPR003583 Hlx-hairpin-Hlx_DNA-bd_motif,	2.7.7.14	Up
5	Nutrient-stress induced DNA binding protein	alr3808	17231300	184	20687.1	5	82.6	IPR002177 DPS_DNA-bd	Protect cells from oxidative and nutritional stresses	Up
**Amino acid biosynthesis**
6	Cysteine synthase [*Nostoc* sp. PCC 7120]	all2521	17230013	319	33653	7.6	110	IPR001926 Cysteine synthase/cystathionine beta-synthase *P*-phosphate-binding site	4.2.99.8	Down
12	D-3-phosphoglycerate dehydrogenase	alr1890	17229382	526	55841.6	5.7	106	IPR006236 D-3-Phosphoglycerate_DH	1.1.1.95	Down
**Unknown proteins**
11	Hypothetical protein all4050	all4050	17231542	324	37259.4	5	104	IPR007903 PRC_barrel		Up
4	Hypothetical protein all3797	all3797	17231289	261	27889.5	9	83.20	IPR000782 FAS1_domain		Up

### PREDICTION AND LOCALIZATION OF FUNCTIONAL DOMAIN, MOTIF ANALYSIS, AND SEQUENCE HOMOLOGY OF HYPOTHETICAL PROTEINS

**FIGURE 3:**
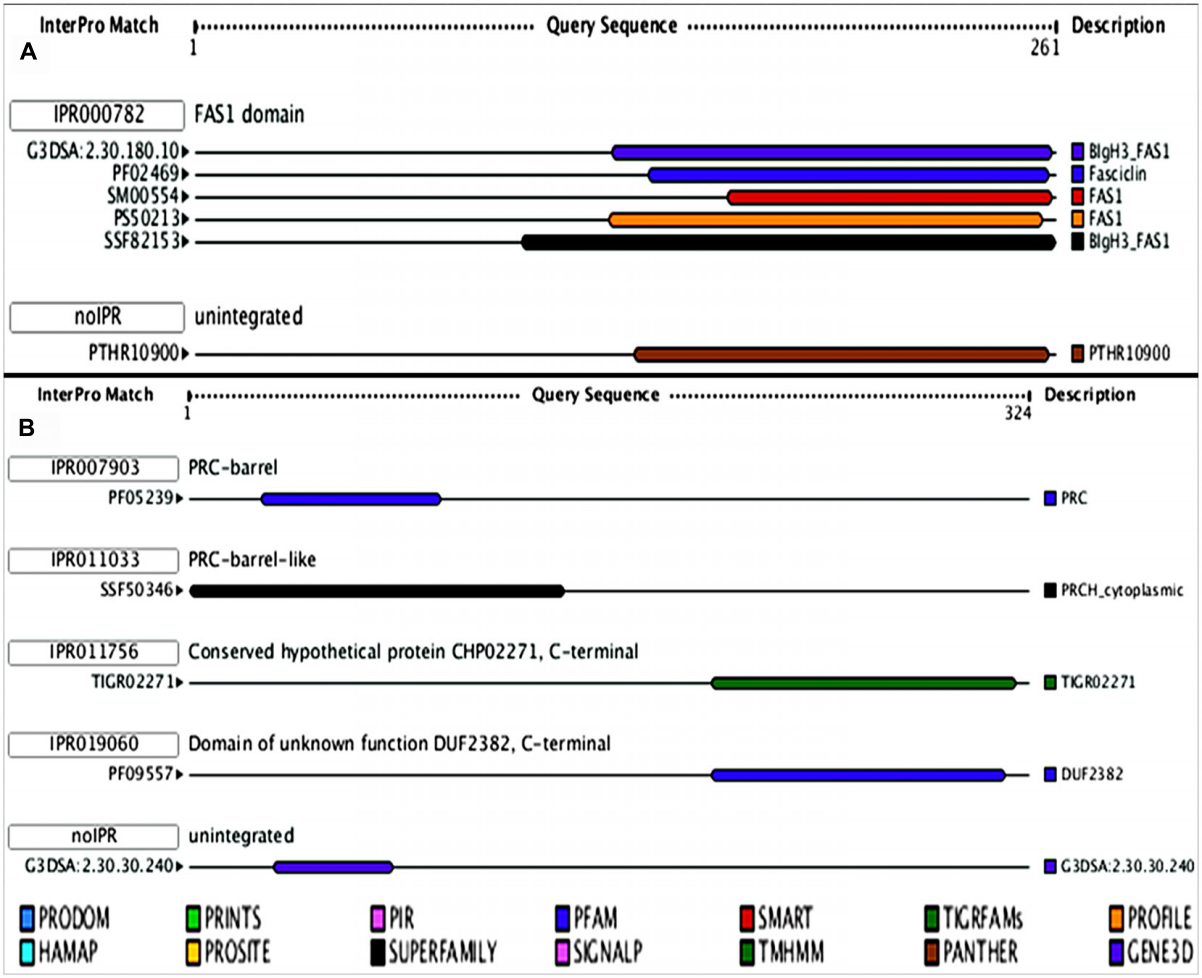
**Model showing the functional domains of hypothetical proteins. (A)**- all3797, and **(B)**- all4050 obtained from (http://www.ebi.ac.uk/Tools/pfa/iprscan/).

As both the HPs (all3797 and all4050) were always induced under UV-B stress and such proteins have not been reported previously from cyanobacteria, we focused our studies on these two proteins. Accordingly, with a view to obtaining preliminary functional information of these two, analysis of functional domain and motif analysis was made. Data obtained revealed that both the proteins possess functional domain as well as regulatory motifs. The protein all3797 encoded by ORF *all3797* showed similarity with the FAS 1 like domain (IPR000782; **Figure 3A**) and all4050 encoded by ORF *all4050* and having four domains (IPR007903, IPR011033, IPR011756, and IPR019060; **Figure 3B**) resembled with the photosynthetic reaction center (PRC).

**FIGURE 4:**
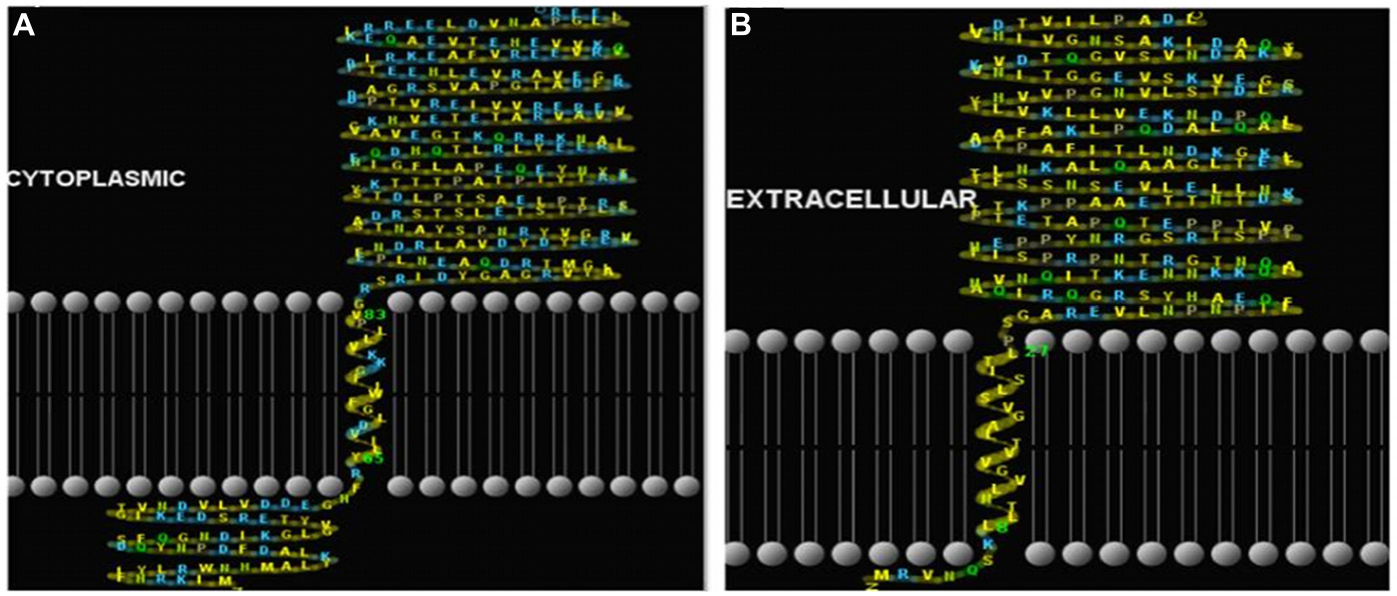
**Prediction of putative signal peptides and transmembrane helices of hypothetical proteins. (A)**-all3797, and **(B)**-all4050 (http://www.cbs.dtu.dk/services/SignalP/) and HMMTop (http://www.enzim.hu/hmmtop/).

Prediction of localization, putative signal peptides and transmembrane helices of the proteins revealed that the protein all3797 has N terminal (inside) with single transmembrane helices from positions 8 to 27 and is extracellularly localized, indicating that it may be a member of type II transmembrane protein family (**Figure 4A**). Protein all4050 showed N terminal (outside) with single transmembrane helices from positions 65 to 83 and is localized in the cytoplasm, implying that it probably be a member of the type I transmembrane protein family (**Figure 4B**).

**FIGURE 5:**
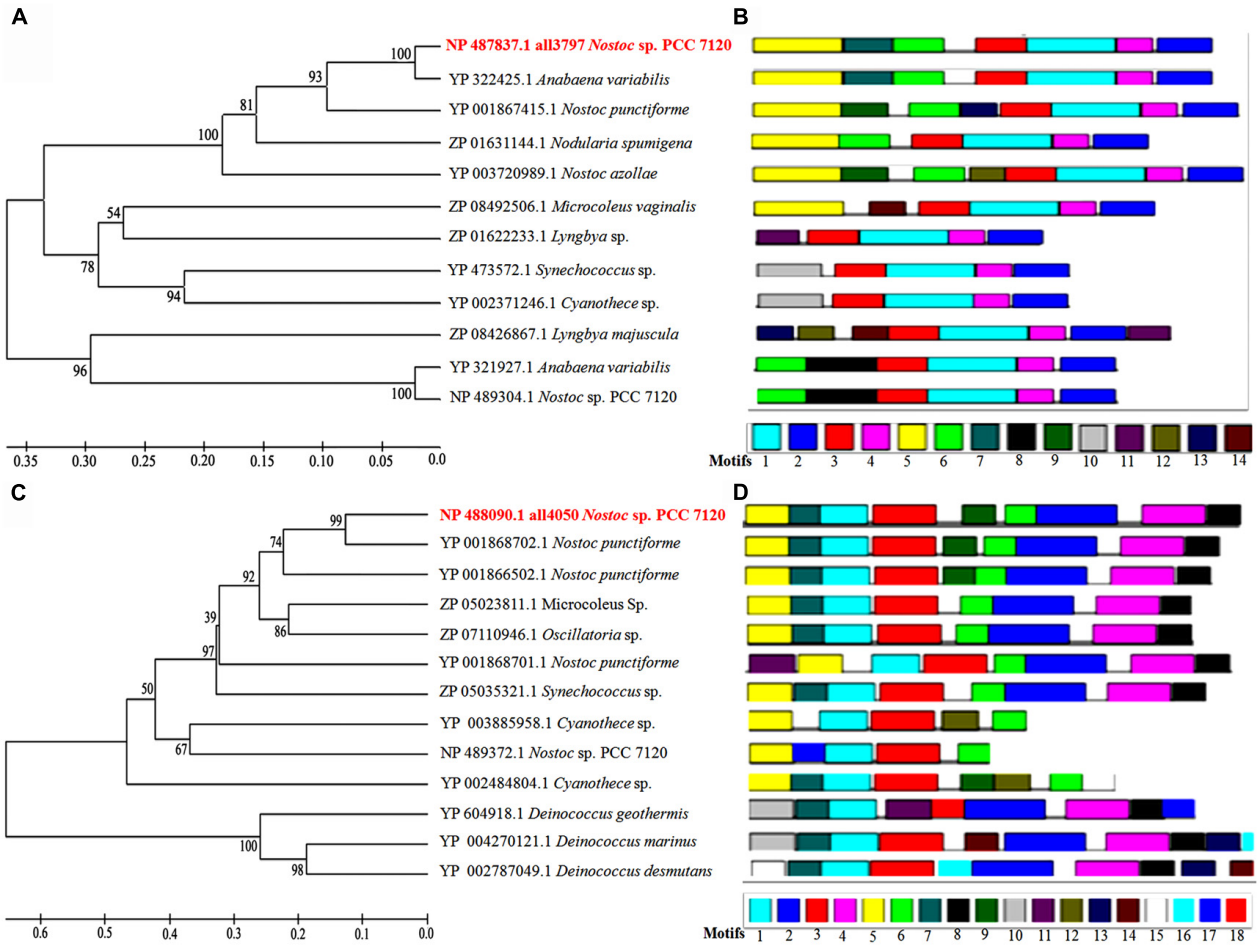
**Phylogenetic tree showing homology with proteins of other species of cyanobacteria and bacteria available in the database**. **(A)**-phyologenetic tree, and **(B)**-motif analysis of all3797; **(C)**-phyologenetic tree, and **(D)**-motif analysis of all4050 (http://www.icp.ucl.ac.be/∼opperd/private/upgma.html) and MEME tool (http://meme.nbcr.net/meme/).

With a view to revealing homology of the proteins all3797 and all4050 with proteins of other species of cyanobacteria (available in data base), a phylogenetic tree was constructed using reference strain *Nostoc* sp. PCC 7120 (also known as *Anabaena* sp. PCC 7120) because of its whole genome sequence availability in database. Accordingly, various cyanobacterial proteins showing more than 30% homology with reference strain were selected for multiple sequence alignment. From the tree it is evident that the protein all3797 shares 100% similarity with the protein of *Anabaena variabilis* (**Figure 5A**). However, a copy of identical ORF is also present in *Nostoc* PCC 7120 but grouped in a separate cluster which might be responsible for certain other functions in this species. HP all4050 showed 99% similarity with the protein of *Nostoc punctiforme* (**Figure 5C**). Analysis of different motifs present in the protein sequences of all3797 suggests that the first four motifs are highly conserved in all the selected species. Furthermore, all the motifs present in all3797 were also present in *A. variabilis* which is in accordance with the similarity index noted in phylogenetic tree (**Figure 5B**). In the case of protein all4050, seven motifs were found to be highly conserved in all the selected species. Interestingly, these motifs were also noted in three species of the bacterium *Deinococcus* (an extreme radioresistant organism) but they also had certain other motifs which resulted in separate cluster formation (**Figure 5D**). It is well-known that the multifunctional consensus sequences corresponding to the motif are useful in understanding the functional aspect of motifs. The sequence analysis shows that in the case of all3797, phenylalanine at position numbers (138, 163, 170), leucine (141,150,154,173,182,183,191), asparagine (246,186), serine (205,245), glycine (247,218,211), threonine (161,166), alanine (144,147,148,164,169), proline (165,174,184), histidine (197,250), glutamate (167), and tyrosine (196) are totally conserved in the sequence alignment. Similarly, in the case of protein all4050, the core conserved residues, namely leucine (16), glycine (50, 61, and 84), arginine (64), and proline (82) were noted.

**FIGURE 6:**
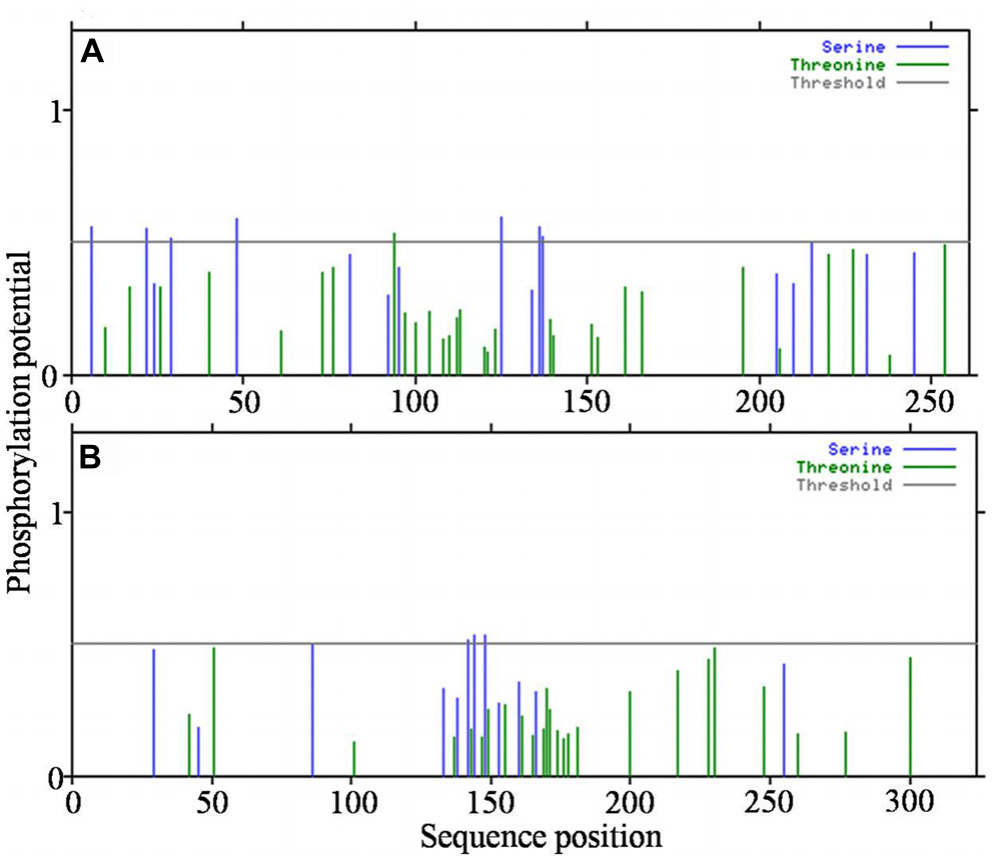
**Detection of post-translational modification sites in hypothetical proteins. (A)**-all3797, and **(B)**-all4050 (obtained from www.cbs.dtu.dk/services/NetPhosBac-1.0/).

Because the analysis of transmembrane helices revealed that both the HPs have one transmembrane helix each, we attempted to detect post-translational modification sites present, if any, in the functional region. Based on the prediction of serine and threonine phosphorylation sites, a total of eight sites were identified in all3797 and three in all4050 (**Figures 6A,B**).

### PREDICTION OF UTR AND PROMOTER REGIONS

As the full genome sequence of *Anabaena* PCC7120 is available in the database^16^, an attempt was made to retrieve upstream region (UTR) of putative genes of both the HPs to make prediction of promoter binding sites and *cis*-regulating elements. Analysis showed that the genes of both the UV-B responsive HPs have promoter regions as well as transcription binding sites in their UTR. Details of promoters and transcription binding sites are presented in **Table 2**. The protein all3797 had promoters argR2 and rpoD17 which are known to play important role against UV radiation and certain other abiotic stresses (**Table 2A**). Likewise, protein all4050 showed the presence of promoters nagC, phoB, rpoD18, and argR whose roles in the regulation of vital metabolic processes are well-documented (**Table 2B**).

**Table 2:** Prediction of UTR and promoter regions of hypothetical proteins all3797 and all4050.

Promoter name	Promoter sequence	Position	Score	Functions
**(A) (all3797)**
**Linear discriminant function (LDF)**
-10 box (2)	TTCTATATT	154	61	
	TTGTAAAAT	560	80	
-35 box (2)	TTGTCA	134	53	
	TTCCAT	538	30	

**TF binding sites**

argR2	CATATTTT	127	7	Regulation of arginine biosynthesis and catabolism. The ArgR protein, a transcriptional repressor, affects the expression level of the *argB* gene through binding to its promoter region.
	CATATTTT	514	8	
fnr	ATCAATTT	143	8	FNR is a global regulator, belonging to the CRP superfamily of helix turn helix DNA binding proteins.
rpoD17	TGTAAAAT	561	11	The “housekeeping” sigma factor or also called as primary sigma factor, transcribes most genes in growing cells. Makes the proteins necessary to keep the cell alive.
	TGTTATAA	568	12	
	AATCTTTA	575	7	
tyrR	TATGTAAC	581	10	Involved in transcriptional regulation of aromatic amino acid biosynthesis and transport.
galR	ATGTAACC	582	7	Repressor of the galactose operon. Binds galactose as an inducer.
deoR	AATTCTAA	591	17	The transcriptional repressor DeoR, for “**D**eoxyribose **R**egulator,” is involved in the negative expression of genes related to transport and catabolism of deoxyribonucleoside nucleotides. Single and double loop formation occur when deoR repressor binds to its natural operator sites.
**(B) all4050**

**Linear discriminant function (LDF)**

-10 box (2)	TTCTATATT	154	61	
	TTGTAAAAT	560	80	
-35 box (2)	TTGTCA	134	53	
	TTCCAT	538	30	
**TF binding sites**
nagC	ATATTTTA	211	7	DNA-binding transcriptional dual regulator, repressor of *N*-acetylglucosamine.
phoB	TTTATTAA	215	8	The positive regulatory gene for the phosphate regulon.
rpoD18	TTTAAAAT	223	7	The “housekeeping” sigma factor or also called as primary sigma factor, transcribes most genes in growing cells. Makes the proteins necessary to keep the cell alive.
argR	TTTTTTAT	246	13	Regulation of arginine biosynthesis and catabolism. The ArgR protein, a transcriptional repressor, affects the expression level of the *argB* gene through binding to its promoter region.

### STRUCTURE MODELING AND STEREOCHEMICAL PROPERTIES

**FIGURE 7:**
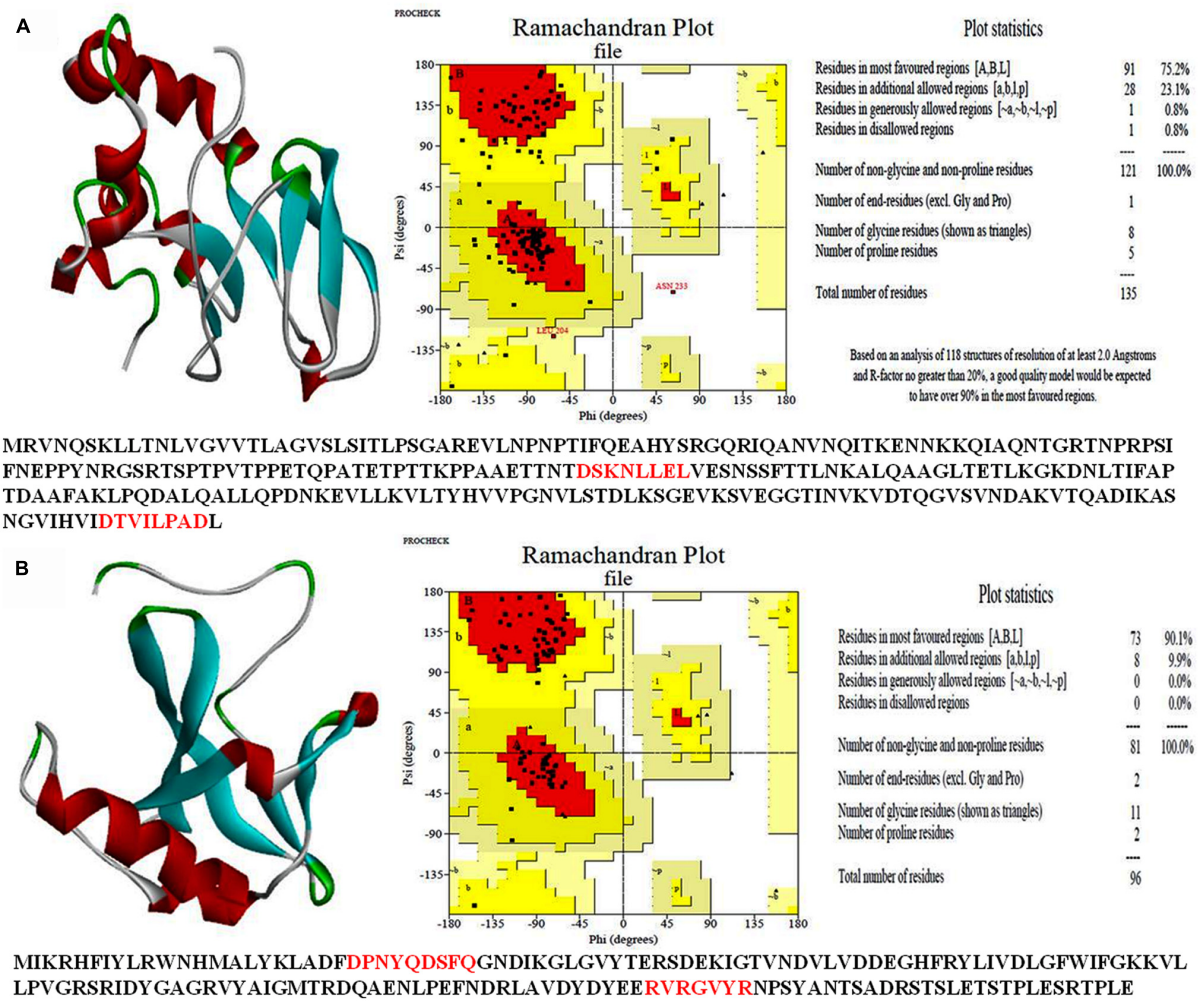
**3-D structure of hypothetical proteins. (A)**-all3797 (PDB ID PM0077736), and **(B)** all4050 (PDB ID PM0077737) obtained from CPHModels (http://www.cbs.dtu.dk/services/CPHmodels/) and PDBSum (http://www.ebi.ac.uk/pdbsum/).

A 3-D model of both the HPs was constructed using sequence similarity and available structures in the data base. Validity of the structure was checked by the Ramchandran plot (**Figures 7A,B**). Such study of the all3797 revealed 75.2% residues in the most favored region, 23.1% in additional allowed region and 0.8% in disallowed region (**Figure 7A**). Similarly, 3-D model of all4050 showed 90.1% residues in the most favored region, 9.9% in additional allowed region and no residue in disallowed region (**Figure 7B**). The values obtained are well within normal range for a high quality model. The best validated structures of all3797 and all4050 have been deposited in the PMDB database with PMDB-IDs; PM0077736 and PM0077737 respectively.

## DISCUSSION

Damaging effects of UV-B radiation on various metabolic processes of different species of cyanobacteria are well-documented (Kumar et al., 2003; Häder et al., 2007; Babele et al., 2012; Singh et al., 2013). In the present study, results obtained showed that UV-B radiation exposure to *Anabaena* L31 in the presence of white light also causes adverse effects on various physiological processes and brings about marked changes in the metabolic machinery. This finding is important and pertinent in view of the fact that living organisms are never exposed to UV-B radiation alone under ambient conditions. Accordingly, to gain better understanding of the metabolic events affected by UV-B stress, we targeted comparative proteomics study of the *Anabaena* L31 cells grown under UV-B stress and normal conditions. As alteration was noted in the expression of several proteins, we selected and identified 21 UV-B responsive proteins whose expression was drastically up- or down- regulated. Alteration in quantity and quality of proteins is expected as it is an essential step of adaptive mechanism developed by cyanobacteria under UV-B stress (Gao et al., 2009). As reported previously (Häder et al., 2007; Singh et al., 2013), that major part of the UV-B energy is absorbed by phycobiliproteins and chlorophyll, these act as the main targets of UV-B action. That phycobiliproteins are indeed the target of UV-B action is supported from our finding wherein the proteins, apcF gene product (all2327), phycocyanin A subunit (alr0529), and phycoerythrocyanin alpha chain (alr0524) are down regulated following UV-B exposure of *Anabaena* L31 cells. This clearly suggests that UV-B absorbed by the cells may directly affect the stability of pigment complex, and prolonged exposure may inhibit the synthesis of phycobiliproteins. Altogether, the damage caused to photosynthetic pigments may lead to diminished PS-II activity resulting in subtle changes in enzymes of Calvin cycle. This is in agreement with an earlier report (Huang et al., 2002) wherein operon composed of genes encoding carbon dioxide-concentrating mechanism proteins (fructose-1, 6-bis phosphate aldolase, glucose 6-phosphate isomerase, and phosphoglucomutase/phosphomannomutase) were found repressed under UV-B stress. Similar to above report, enzymes such as fructose-1, 6-bisphosphate aldolase (all4563), and glucose 6-phosphate isomerase (alr1050) were down regulated under UV-B stress in our study. Fructose-1, 6-bis phosphate aldolase is an important enzyme of glycolysis and Calvin cycle that converts fructose-1, 6-bis phosphate to glyceraldehyde 3-phosphate and dihydroxy acetone 3-phosphate. Under stress conditions glyceraldehyde 3-phosphate and fructose-1, 6-bis phosphate may be converted to glucose 6-phosphate to carry out pentose phosphate pathway for NADPH synthesis (Nakahara et al., 2003). This is an important step as it may partly meet the reductant requirement for vital metabolic processes. Down regulation of phosphoglucomutase (all3964) following exposure of *Anabaena* L31 cells to UV-B is an interesting observation and to our knowledge not reported from any other species of cyanobacteria. Phosphoglucomutase may have significant role under UV-B stress as it converts glucose-1-phosphate to glucose -6-phosphate which is an important reversible reaction of glycolysis (Fridlyand and Scheibe, 1999).

That energy metabolic proteins were one of the smallest fractions of the identified proteome in this study is in agreement with the report that majority of these proteins remain embedded into integral membranes and only a few occur in soluble fraction (Anderson et al., 2006). Consistent with the down regulation of proteins of carbohydrate metabolism, the transcript allowing ATP synthesis by F0F1 ATP synthase (all5039) was down regulated in the presence of UV-B stress. This enzyme is of great significance to the test cyanobacterium as it uses a proton gradient to drive ATP synthesis and hydrolyzes ATP to build the proton gradient. Another important protein identified as phosphoribulokinase (alr4123) which catalyzes the ATP-dependent phosphorylation of ribulose-5-phosphate to ribulose-1, 5-phosphate during CO_2_ assimilation by autotrophic organisms was also down regulated under UV-B stress. This may also adversely affect pentose phosphate pathway and growth of the test organism.

Amino acids are essential components of proteins with a key role in protein biosynthesis and regulation of growth and development of all living organisms. In this context, our data show that D-3-phosphoglycerate dehydrogenase *(*alr1890) and cysteine synthase (all2521) are down regulated under UV-B stress and thus may adversely affect the amino acid biosynthesis pathway. Down regulation of D-3-phosphoglycerate dehydrogenase which catalyzes the formation of 3-phosphonooxypyruvate from 3-phospho-D-glycerate in the serine biosynthesis may reduce the level of α-ketoglutarate and affect the formation of 2-hydroxyglutarate. Likewise, down regulation of cysteine synthase may also affect the synthesis and cellular level of L-cysteine as it converts *O*_3_-acetyl-L-serine into L-cysteine. Conceivably, diminished growth of *Anabaena* L31 under UV-B stress could be due to reduction in the level of enzymes of amino acid biosynthesis.

Among the up-regulated proteins, those involved in DNA repair, chaperonins, and antioxidative defense mechanism are the most prominent. Induction of proteins involved in DNA repair such as nutrient stress-induced DNA-binding protein (alr3808) and DNA-directed RNA polymerase alpha subunit (all4191) suggests that DNA could be a target of UV-B effects in *Anabaena* sp. This is also supported from our earlier conclusion where we reported that that UV-B affects the DNA of cyanobacteria and the killing of these microbes might be due to the irreversible damage caused to DNA by this high energy radiation (Kumar et al., 2004). To this effect, up-regulation of DNA-binding protein is expected as DNA-binding protein from stationary phase (DpsA) of the caynobacterium *Synechococcus* sp. PCC7942 is known to confer resistance to oxidative stress and long-term nutrient starvation (Pena and Bullerjahn, 1995; Sen et al., 2000). A DpsA homolog has also been reported from *Nostoc* (*Anabaena*) sp. PCC7120 (Hernandez et al., 2007). To our knowledge, there is no report showing induction of DNA-binding protein under UV-B stress in any cyanobacteria; so identification of alr3808 protein in this study may be a novel finding. RNA polymerases that make up transcription machinery routinely traverse through many parts of the genome; this transcription machinery seems to have an important role in sensing DNA repair and stress responsive pathway (Ljungman, 2007). As DNA-directed RNA polymerase and nutrient-induced DNA-binding proteins are regulated by the transcription machinery, their up regulation may be a common event under different abiotic stresses.

Various abiotic stresses can induce chaperonin GroEL, including heat, acid, radicals, and UV radiation (Frydman, 2001; Jang et al., 2004; Bukau et al., 2006; Gao et al., 2009). We also observed four-fold up regulation of GroEL (all3662) and GroES (alr3661) in response to UV-B stress in *Anabaena* L31. Similar response of above proteins in plants and bacteria under stress condition has been reported (Jang et al., 2004). It has been suggested that proteomic mechanisms act as a check point inside the cell under stress conditions with crucial roles in modifications and survival of cyanobacteria (Balogi et al., 2008; Gao et al., 2009). The most striking feature of the proteins involved in protection against oxidative stress is their expression and maintenance of homeostasis in a stressed environment. In this context the activated antioxidative systems under stress play key role in the protection mechanism of any organism. In the present study, iron superoxide dismutase (SOD; alr2938) and 1-cys peroxiredoxin (alr4404) increased fourfold following UV-B stress and could possibly mitigate the damaging effect of increased ROS level in the cells. SOD effectively reduces superoxide radicals to hydrogen peroxide and peroxiredoxins metabolize peroxides. Peroxiredoxins have multiple roles including reduction of H_2_O_2_ and organic hydrogen peroxides (Dietz et al., 2006), and as molecular chaperones similar to HSPs with roles in cell signaling (Jang et al., 2004).

Inorganic-pyrophosphatase plays a critical role in lipid metabolism (including lipid synthesis and degradation) and DNA synthesis, as well as in other biochemical transformations. By promoting rapid hydrolysis of pyrophosphate (PPi), inorganic pyrophosphatase activates oxidation of fatty acids. As UV-B radiation is reported to cause lipid peroxidation (Rai et al., 2013), over-expression of inorganic pyrophosphatase in this study might be due to this stress.

One novel finding from this study is the identification and characterization of two proteins (all3797 and all4050) annotated as HPs which are new and so far not reported from any cyanobacteria subjected to UV-B stress. *In silico* analysis revealed that all3797 is related to the FAS1 (fasciclin-like) domain and is an extracellular module of about 140 amino acid residues of fasciclin superfamily protein. It seems to be involved in cell adhesion with extracellular matrix and may be responsible for aggregation of cyanobacterial filaments to protect them from UV radiation.

Another HP all4050 showed similarity to PRC (barrel)-like protein. The PRC-barrel, approximately 80 residues long, is widely distributed in bacteria, archaea and plants. Its role in a variety of biological systems, ranging from RNA processing to photosynthesis is well-documented. Several researchers have reported drastic effects of UV-B irradiation on the water soluble pigment phycobiliprotein, including disintegration of phycobilisomes and loss of α and β monomers of phycocyanin. Additionally, phycobiliproteins act as photosensitizers and produce ROS under UV-B stress. Most probably enhanced expression of PRC-barrel protein all4050 may be the result of stress caused by UV-B and may partially restore light-driven electron transfer reactions, further study is needed to reveal the exact function of all4050 under UV-B stress. However, we have provided sufficient data related to localization, functional domain, signal peptide, sequence similarity, motif types and UTR and promoter regions of both the HPs by applying *in silico* approaches. Prediction of functional domains, UTR, motif analysis, and putative model of these HPs could be useful in future studies dealing with unknown proteins specifically induced under other types of abiotic stress. In summary, the protein profiling and expression data obtained in this study suggest that UV-B stress is quite effective in the presence of white light but more work is warranted to unravel the complexity of the proteome following growth of cyanobacteria under different light regimes with UV-B radiation.

## CONCLUSION

A few researchers have reported changes in the proteome of cyanobacteria under UV-B radiation stress but those studies were mostly conducted on the unicellular cyanobacterium, *Synechocystis* sp. PCC 6803 but only in the presence of UV-B light. Our study is the first of its kind that demonstrates changes in the proteome of the N_2_-fixing *Anabaena* strain L31 following UV-B radiation stress in the presence of white light. Analysis of 223 proteins on 2-D gels revealed several differentially expressed proteins of which 21 spots were identified by mass spectrometry. Among the identified proteins, chaperonin GroEL and groES gene product, 30S ribosomal protein S1, and iron SOD are the well-documented UV-B induced proteins but induction of certain novel proteins including HPs (all3797 and all4050) under UV-B stress is new and not reported so far. It would be worthwhile to make detailed and comparative analysis of UV-B responsive proteins from both non-N_2_-fixing and N_2_-fixing cyanobacteria to better understand the stress tolerance mechanism against UV-B as well as the roles of different classes of proteins in this important process. Further study is needed to assign the exact function of HPs which are specifically induced under UV-B stress. Nevertheless, identification of differentially expressed proteins in this study may prove useful in future studies especially for assessing their significance in the adaptation mechanism of cyanobacteria under UV-B radiation stress.

## Conflict of Interest Statement

The authors declare that the research was conducted in the absence of any commercial or financial relationships that could be construed as a potential conflict of interest.

## Footnotes

1 http://www.matrixscience.com

2 http://web.expasy.org/protparam/

3 http://www.genome.jp/tools/blast/

4 http://biocyc.org/blast.html

5 http://www.genome.jp/kegg/genes.html

6 http://www.cbs.dtu.dk/services/SignalP/

7 http://www.enzim.hu/hmmtop/

8 http://www.ebi.ac.uk/Tools/pfa/iprscan/

9 http://blast.ncbi.nlm.nih.gov/Blast.cgi

10 http://meme.sdsc.edu/meme/intro.html

11 http://genome.kazusa.or.jp/cyanobase

12 http://linux1.softberry.com/berry.phtml

13 http://www.cbs.dtu.dk/services/NetPhosBac-1.0/

14 http://www.cbs.dtu.dk/services/CPHmodels/

15 http://www.ebi.ac.uk/pdbsum/

16 http://genome.kazusa.or.jp/cyanobase/Anabaena
